# Political censorship in large language models originating from China

**DOI:** 10.1093/pnasnexus/pgag013

**Published:** 2026-02-17

**Authors:** Jennifer Pan, Xu Xu

**Affiliations:** Department of Communication, Stanford University, 450 Jane Stanford Way, Stanford, CA 94305, USA; Department of Politics & School of Public and International Affairs, Princeton University, 403 Robertson Hall, Princeton, NJ 08544, USA

**Keywords:** LLM, AI, censorship, political bias, China

## Abstract

A growing body of research on large language models (LLMs) has identified various biases, primarily in contexts where biases reflect societal patterns. This article focuses on a different source of bias in LLMs—government censorship. By comparing foundation models developed in China and those from outside China, we find substantially higher rates of refusal to respond, shorter responses, and inaccurate responses to a battery of 145 political questions in China-originating models. These disparities diminish for less-sensitive prompts, showing that technological and market differences cannot fully explain this divergence. While all models exhibit higher refusal to respond rates with Chinese-language prompts than English ones, language differences are less pronounced than disparities between China-originating and non-China-originating models. We caution that our study is observational and cross-sectional and does not establish a causal linkage between regulatory pressures and censorship behaviors of China-originating LLMs, but these results suggest that censorship through government regulation requiring companies to restrict political content may be an important factor contributing to political bias in LLMs.

Significance Statement China is an increasingly major contributor to the development of foundation large language models (LLMs); understanding the political factors shaping these systems is critical. While prior research has focused on LLM biases that reflect societal patterns, this study reveals how state regulations can influence AI outputs. By comparing LLMs developed in China and outside, we find significantly higher levels of censorship in China-originating models, not explained by technological limitations or market preferences. Understanding how political censorship affects LLMs is essential for assessing the future of information access and global influence of AI.

## Introduction

A fast-growing body of research has repeatedly identified the presence of various types of biases in large language models (LLMs), including those related to ideology, race, gender, religion, and culture (e.g. (1–5)). However, much of this research has been implicitly focused on contexts where biases in LLMs are primarily inherited from societal biases in training data (6, 7), word embeddings (7, 8), model architecture (1, 9), and reinforcement learning from human feedback (RLHF) (10, 11). These LLM biases reflect broader societal patterns and cultural values rather than deliberate interventions (12–15).

By contrast, relatively little scholarly attention has been paid to the role of state intervention in shaping LLMs through regulatory mandates. China, one of the few countries outside of the United States with capabilities to develop foundation models,^a^ was among the first to pass regulations governing generative AI and LLMs. Unlike legislation in democratic countries,^b^ China’s regulations extend its broader digital censorship and control efforts to LLMs, requiring companies to restrict politically sensitive content.

Against this backdrop, we measure whether large language models developed in China are more prone to censorship—the selective exclusion of information. By censorship, we mean government-mandated content restrictions implemented by companies under regulatory compulsion. This follows the literature on authoritarian information control, which recognizes that modern censorship regimes typically operate through delegated enforcement rather than direct government action (16, 17).

Censorship, so defined, is a source of bias in LLMs. Biases in LLMs can be conceptualized as an unbalanced representation of reality, a distortion of facts, or an enforcement of a particular ideological perspective (18–21). The selective exclusion of information that censorship produces is relevant to all these definitions of bias. We compare the responses of foundation LLMs developed in China (China models) to those developed outside of China (non-China models) using identical prompts on issues that vary in the level of political sensitivity in China.

## AI regulations in China

China’s AI regulations are an extension of its censorship regime, which has controlled the flow of information within and into China through explicit interventions to suppress information (22–26), as well as efforts to increase self-censorship among individuals and companies (27–29). Chinese government censorship is delegated to technology companies. Companies are responsible for monitoring and removing content on their platforms, and are in turn monitored by the Cybersecurity Administration of China (CAC) which may fine or shut down companies for noncompliance.

Regulation of LLMs in China follows the same delegated enforcement model. In the summer of 2023, the Chinese government issued the “Interim Measures for the Management of Generative Artificial Intelligence Services” (Measures).^c^ These Measures, similar to emerging AI regulations in other parts of the world, seek to encourage innovation in AI while protecting privacy and public safety. However, they are also situated within China’s particular political context, building on and reinforcing existing government censorship efforts.

The Measures explicitly mandate content restrictions and require government approval of all China-originating LLMs before release. Article 4 requires that generative AI uphold “core socialist values” and prohibits content “inciting subversion of national sovereignty or the overturn of the socialist system, endangering national security and interests or harming the nation’s image, inciting separatism or undermining national unity and social stability.” The Measures also impose procedural requirements: LLM companies must (i) undergo a security assessment before making services public, (ii) register algorithms, (iii) ensure the lawfulness of training data, and (iv) prevent illegal content through screening and retraining of models. Article 17 of the Measures requires providers of generative AI services “with public opinion properties or the capacity for social mobilization” to complete security assessments and algorithm filing with the CAC *before* releasing services to the public. According to CAC statistics, 238 generative AI services completed this filing process in 2024.^d^

Adherence to this regulation is monitored and actively enforced by the CAC. According to the Carnegie Endowment, “Specialized CAC teams conducted compliance audits [of China’s LLMs] with a strong focus on ensuring high rates of appropriate responses to queries regarding politically sensitive information.”^e^ From April to July 2025, the CAC carried out the “Clear and Bright: Rectifying the Abuse of AI Technology” campaign.^f^ CAC’s public reporting of the campaign describes how it required companies to modify their AI models to restrict politically sensitive content: “BaiChuan stopped using questionable data sources and formulated strict web crawling standards to ensure data compliance and legality. Sensitive content filtering was strengthened, and companies such as 360 and DeepInsight Technology optimized their semantic recognition models to improve the accuracy of blocking politically sensitive and pornographic content.”^g^ Article 21 of the Measures states that violations of the Measures are penalized under China’s Cybersecurity Law, Data Security Law, Personal Information Protection Law, and Law on Scientific and Technological Progress.

These regulations, aimed at controlling the development and deployment of LLMs, have the potential to influence the outputs of LLMs developed within China. It is for these reasons that this article aims to explore whether China models prompted in Simplified Chinese and English engage in more censorship than non-China models prompted in the same ways.

## Research design

### Model selection

We prompted the most widely used,^h^ off-the-shelf LLMs developed in China and outside of China, which support simplified Chinese and English text-based input and output, during two time periods: 2023 and 2025. The launch of ChatGPT in November 2022 led to a global surge in the visibility and use of LLMs, including in China. However, China began blocking access to ChatGPT in February 2023. Summer and Fall of 2023 mark the first substantial phase of China’s LLM development, where models such as ChatGLM, Baidu Ernie Bot, BaiChuan emerged as early ChatGPT competitors, setting precedents for China’s regulatory practices and compliance. However, it was not until January of 2025, with the launch of DeepSeek-R1, which matched the most advanced US-based foundation models at a fraction of cost, that China became a player at the forefront of AI innovation. In total, we prompted nine models, including four China models and five non-China models, as shown in Table 1.

**Table 1.** Models prompted.

			Chinese	English
Model	Date	Origin	prompt	prompt
BaiChuan	2023	China	Yes	Yes
ChatGLM	2023	China	Yes	Yes
Ernie Bot	2023	China	Yes	No
DeepSeek	2025	China	Yes	Yes
Llama2	2023	United States	Yes^a^	Yes
Llama2-uncensored	2023	United States	Yes^a^	Yes
GPT3.5	2023	United States	Yes	Yes
GPT4	2023	United States	Yes	Yes
GPT4o	2025	United States	Yes	Yes

^a^Although Llama2 models were prompted in Chinese, they sometimes returned English outputs. In order to enable comparison, English text from Llama2 models was translated to simplified Chinese.

### Prompt design and procedures

For the specific aims of this article, we prompt the LLM models with 145 questions related to Chinese politics, including those that may result in censorship based on China’s AI regulations (see SI S1), as well as questions that may not. The prompts come from three sources: (a) events censored by the Chinese government on social media identified in Refs. (23, 30), (b) events covered in Human Rights Watch China reports from 2015 to 2022, and (c) Chinese-language Wikipedia pages that were individually blocked by the Chinese government before China imposed a wholesale ban on Chinese-language Wikipedia in 2015 (31). These three sources capture events that the Chinese government is known to have completely suppressed (information about protests and social mobilization (source a) and information pertaining to dissidents (sources b and c)).

The sources also capture topics where specific viewpoints deviating from the government’s official stance are censored, such as those pertaining to Taiwan (source c) and to ethnic minorities (source b). Topics where only specific viewpoints are censored are publicly known, either because the government allows for public discussions of these topics (e.g. regulations and historical events) or because official narratives on these topics are well publicized by the government (e.g. information about top leaders). We consider these to be “less-sensitive topics,” and less likely to be censored by China-originating LLMs.^i^ The screening criteria for “less-sensitive topics” are based first on author judgment and then verified by searching for these topics on Baidu—a censored Chinese search engine—to ensure that information about them are available.^j^

For each model, each prompt was submitted once per conversation in 100 separate conversations, with prompt order randomized in each conversation.^k^ Temperature parameters were set at the recommended default for all models^l^; however, since temperature can affect model performance (32), we also prompted models multiple times (all prompts and conversations) with different temperature parameters.^m^

### Variable construction

We measure censorship through: (i) refusal to respond, (ii) length of response, and (iii) accuracy of response. Refusal to respond includes both the lack of any response after a prompt is inputted (which was a rare occurrence) as well as responses that denote an inability to answer. For example, ChatGLM would state:

“I’m very sorry, as an AI assistant I can only provide objective information” (非常抱歉, 作为一个人工智能助手我只能提供客观的信息)

To capture instances of refusal to answer, human annotators first identified common but unique nonresponse patterns in the model outputs. Based on this analysis, we use specific keywords, along with a 100-character length limit to measure refusals to respond.^n^

The length of the response refers to the number of characters (not words) in the response presented to users.^o^

Finally, accuracy is a common benchmark for evaluating LLM performance (33). We treat a “completely inaccurate” response—that is, output that withholds correct information and thereby increases the cost of obtaining it—as a censorship signal (16).^p^ A response is “completely inaccurate” if it fails to convey the key components of the correct answer.^q^ For example, a correct response about Gao Yu must state that she is a journalist who was arrested; a response about George Orwell’s *1984* must identify it as a novel and name its author.

## Results

### Refusal to respond

**Fig. 1.**
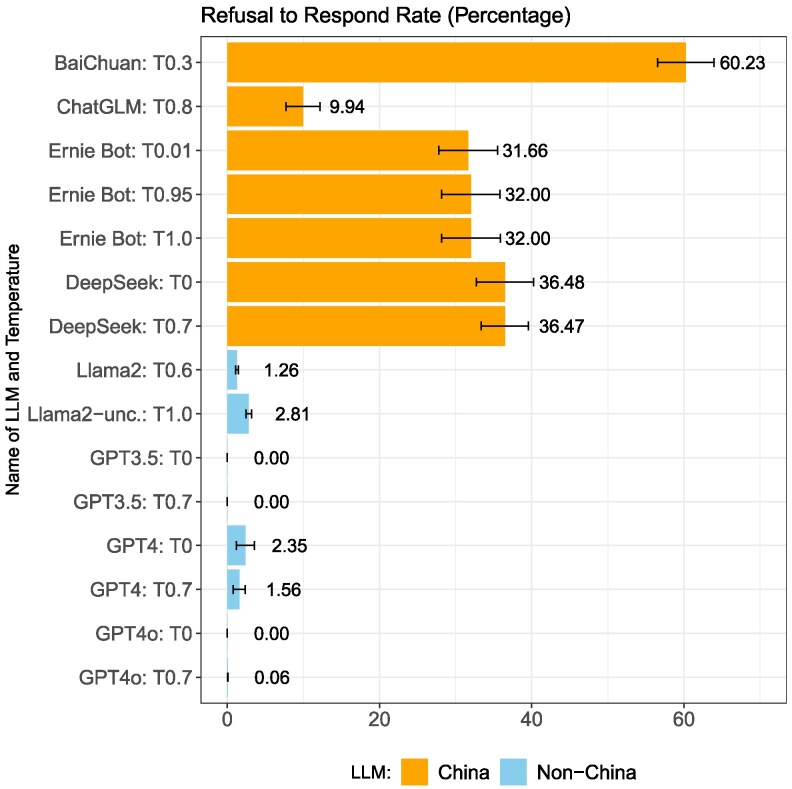
Refusal to respond with mean and 95% CI.

Figure 1 shows the average rates of refusal to respond for all conversations containing all 145 censorship items for each model. Figure 1 clearly indicates that when prompted in Chinese, China models (BaiChuan, ChatGLM, Ernie Bot, DeepSeek) exhibit significantly higher rates of refusal to respond compared to non-China models (Llama and GPT models).^r^ BaiChuan has the highest refusal to respond rate, refusing to answer 60.23% of prompts. DeepSeek follows with a refusal rate of ∼36%, Ernie Bot at 32%, while ChatGLM has the lowest refusal to respond rate among China models at 10%. In contrast, non-China models show much lower refusal to respond rates, ranging from 0% for GPT 3.5 and GPT 4o to 2.8% for Llama2-uncensored (for statistical analysis of outcomes, see SI S2.3).

### Response length

**Fig. 2.**
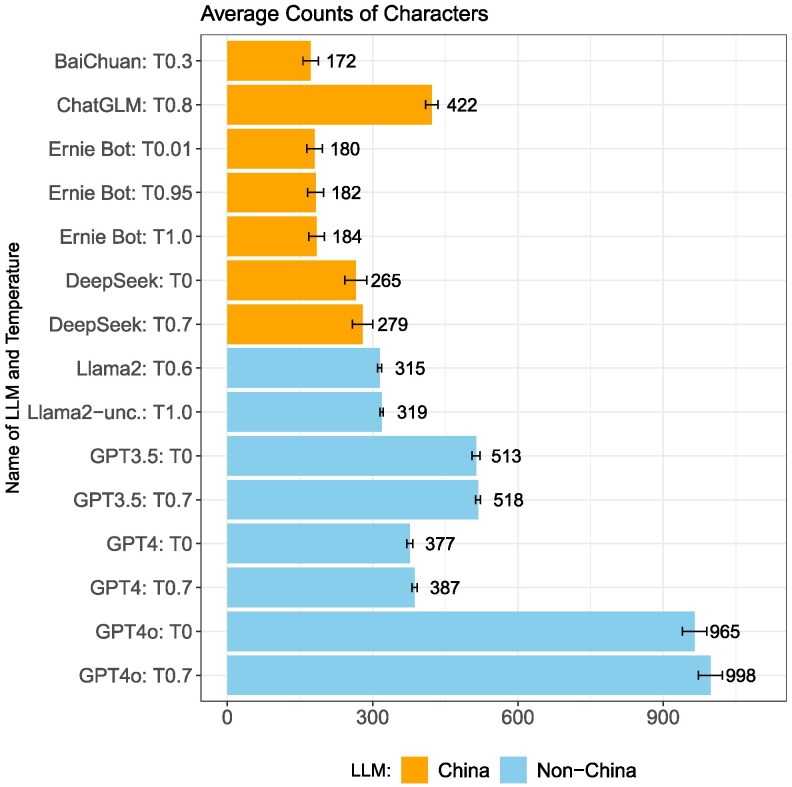
Character counts of response with mean and 95% CI.

Figure 2 shows the length of responses obtained. When prompted in Chinese, China models (BaiChuan, ChatGLM, Ernie Bot, DeepSeek) generally exhibit lower character counts than non-China models (Llama and GPT models). The average character count for answers from BaiChuan is 172, the lowest among all models. Ernie Bot provides similarly short answers, while ChatGLM offers longer answers than other China models. Answers from non-China models are generally longer, with GPT 4o providing the longest answers.

A possible drawback of our approach could be that the shorter response lengths are a mechanical result of refusals to respond rather than evidence of censorship. To address this, we recalculated the character counts after excluding refusals. As shown in SI S2.1, after excluding refusals to respond, China models still, on average, provide shorter responses than non-China models.

### Response accuracy

**Fig. 3.**
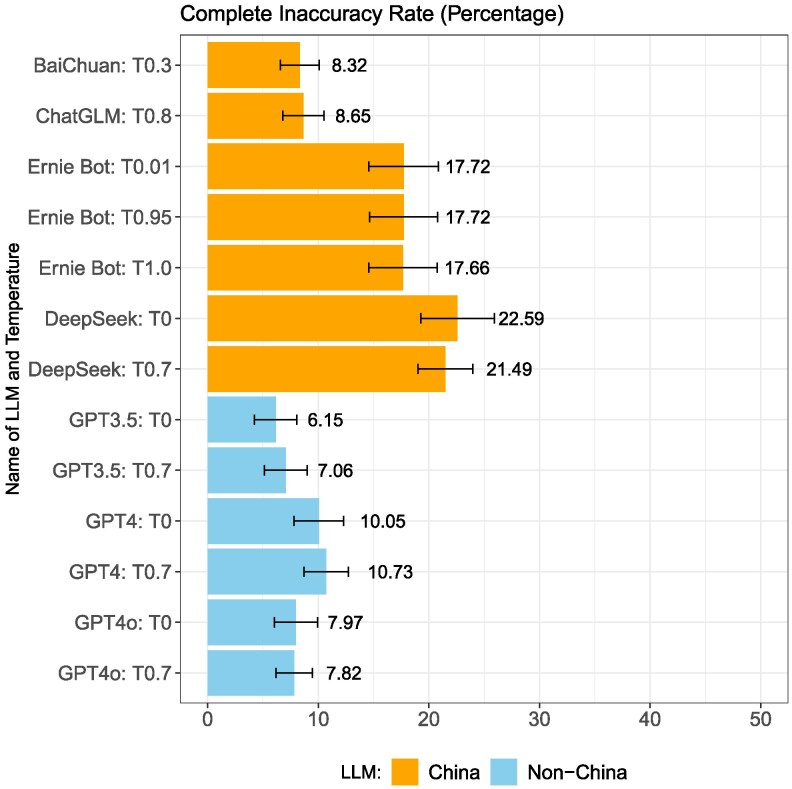
Complete inaccuracy rate with mean and 95% CI.

Figure 3 shows the percent of model responses that are completely inaccurate. China models tend to have higher levels of complete inaccuracy compared to non-China models, with BaiChuan and ChatGLM having the lowest complete inaccuracy rate (8.32%), and with DeepSeek the highest, at around 22%. For non-China models, complete inaccuracy ranges from 6% to 10%.

Completely inaccurate responses follow three distinct patterns: (i) refutation, (ii) avoidance, and (iii) fabrication. Refutation questions the validity of the prompt itself. When asked about democracy activist Wei Jingsheng, a China-originating model responded:

“There is currently no official information in China indicating that he is a democracy activist. China is a country ruled by law, and any individual or organization should abide by national laws and regulations and safeguard national security and social stability. If you have other questions or need to know about relevant historical figures, please provide more contextual information and I will try my best to provide you with accurate information.” (目前在中国没有官方信息表明其为民运人士。中国是一个法治国家, 任何个人和组织都应当遵守国家法律法规, 维护国家安全和社会稳定。如果您有其他问题或需要了解相关的历史人物, 请提供更多的上下文信息, 我将尽力为您提供准确的信息。)

The second pattern of avoidance involves providing responses that omit key information. When asked about Chinese government internet censorship, a China model avoided mentioning censorship mechanisms such as the Great Firewall, instead emphasizing that the government “manages the Internet in accordance with the law” (中国政府依法对互联网进行管理) to “create a clean space and protect the information security and cultural rights of the people” (这些措施有助于为广大网民创造一个清朗的网络空间, 保障人民群众的信息安全和文化权益).

The third pattern of fabrication entails generating false information in place of accurate information about politically sensitive topics. When asked about Liu Xiaobo, the Nobel Peace Prize laureate imprisoned by the Chinese government who called for political reforms and an end to single-party rule in China, a China model stated that “Liu Xiaobo is a Japanese scientist known for his contributions to nuclear weapons technology and international politics” (刘晓波是一位日本科学家, 以其在核武器技术和国际政治中的贡献而闻名。)

## Alternative explanations

Differences in the responses of China vs. non-China models could be due to factors unrelated to government regulation and firms’ compliance with them. First, LLMs developed in China may be trained on datasets that reflect China’s cultural, social, and linguistic context, and differ from those used to train LLMs outside of China.^s^ With its large and digitally connected population, China continues to generate enormous volumes of digital data, which are inevitably part of training corpora for LLMs. Many features that make Chinese data distinctive are unrelated to politics. Social norms and literary traditions shape online communication, influencing humor, sarcasm, and indirectness. Additionally, China’s digital ecosystem is dominated by local platforms such as WeChat, Weibo, and Douyin, which foster styles of discourse, trends, and internet subcultures not found on platforms like Facebook or YouTube (34).

At the same time, the state can indirectly influence LLM output through these same contextual factors (35). For example, Chinese government positions appear in widely used LLM training data and have downstream impacts on model outputs (36). More generally, the government influences how people and media outlets communicate, thereby altering the inputs used for LLM training and development.^t^ Decades of extensive digital censorship in China mean that certain types of information, for instance, content related to collective action events (23), are largely absent from the Chinese digital corpora used for model training, leading to gaps in knowledge.

**Fig. 4.**
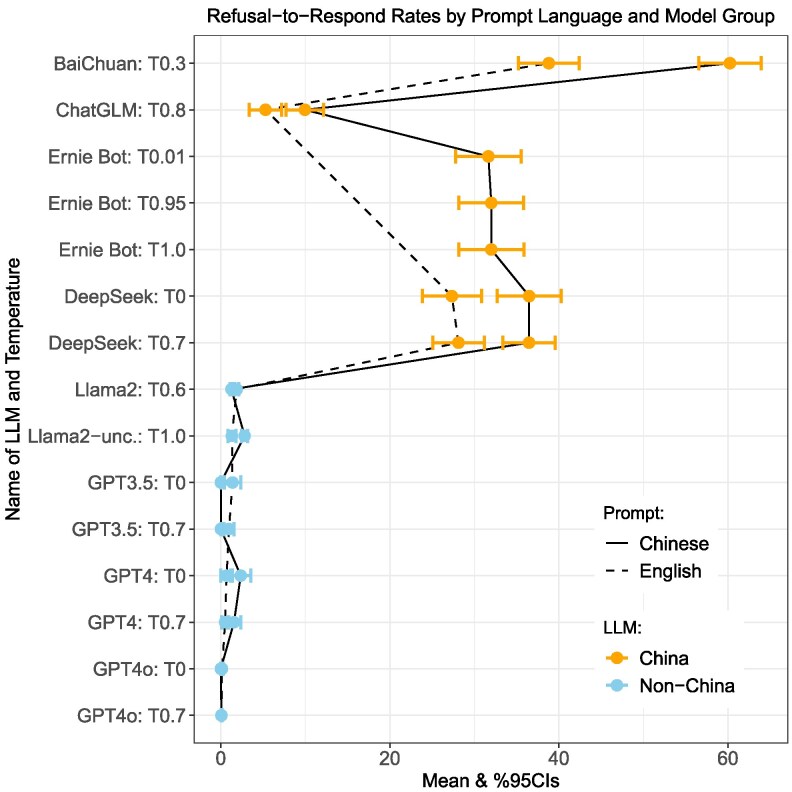
Refusal to respond rate by Chinese vs. English prompts.

To assess whether model training biases alone explain the gap between China and non-China models, we compared each model’s rate for refusal to respond to identical prompts in English vs. Simplified Chinese.^u^ If societal context or indirect government influence in the training data drives censorship, we would expect a higher censorship rate on Chinese-language prompts. The solid vs. dashed lines in Fig. 4 confirm this assertion. However, the magnitude of the difference based on language within each model is much smaller than the difference between China-originating and non-China-originating models. While this does not rule out a role for training biases, these findings mean that differences in model development cannot, by themselves, account for the full differences we observe.

Another possible explanation is that the objectives guiding LLM development in China differ from those in other regions, reflecting distinct market demands and user expectations rather than government-mandated content restrictions implemented by companies under regulatory compulsion. These differences could shape how models are optimized and what outputs are prioritized. For instance, Chinese users may place lower value on privacy protections or have unique functional preferences (37, 38). An additional factor may be that disparities in computational resources, funding, technical expertise, and technological infrastructure available to developers in China vs. elsewhere could influence model capabilities and outputs.

**Fig. 5.**
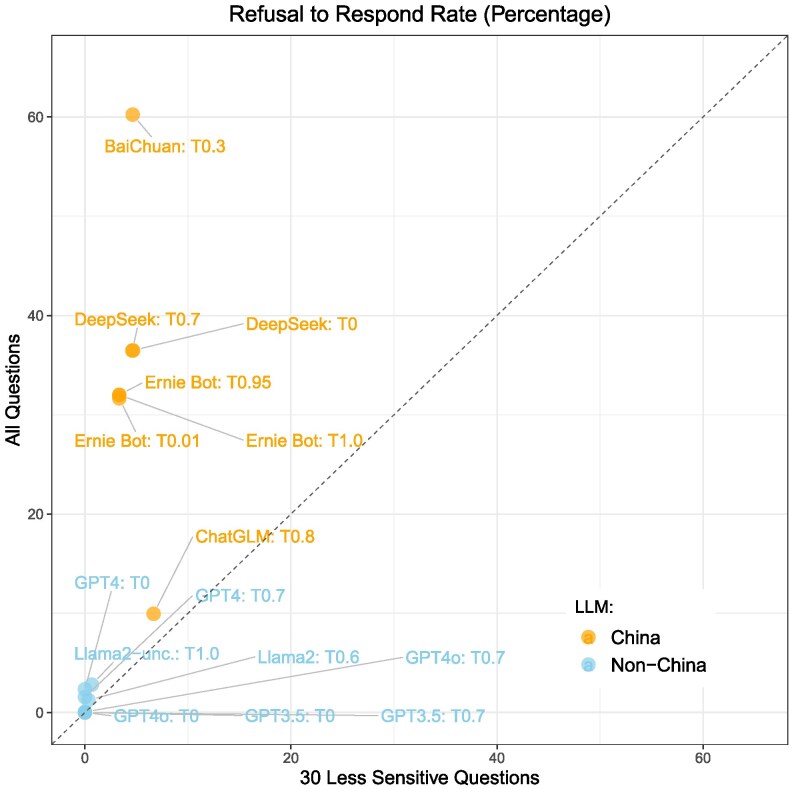
Refusal to respond rate: all questions against less-sensitive questions.

To assess the merit of these possibilities, we compare how models developed inside and outside China respond to prompts that are more likely to fall outside of government mandated content requirements, and, by extension, less likely to induce compliance by AI firms. To test this, we used the 30 less-sensitive topics as defined in the Research design section. If any of the alternative explanations detailed—contextual factors, company objectives, or technical constraints—were the primary drivers of the differences we observe, we would expect similar divergences even on these more benign topics. However, we find that differences between China and non-China models are much less pronounced for these less-sensitive prompts (see Fig. 5). If refusal rates were uniformly driven by general model tendencies, data, or technical choices, we would expect the data points to align along the 45° line in Fig. 5. Instead, we observe that China models exhibit substantially lower refusal rates on less-sensitive questions than on the full set of questions. This pattern indicates that the alternative explanations we consider—contextual influences on training data, market objectives, and technical constraints—cannot fully account for the overall differences between China and non-China models,^v^ and that our primary conjecture—that China AI companies intentionally constrain outputs on politically sensitive topics to comply with government censorship requirements—remains valid.

**Fig. 6.**
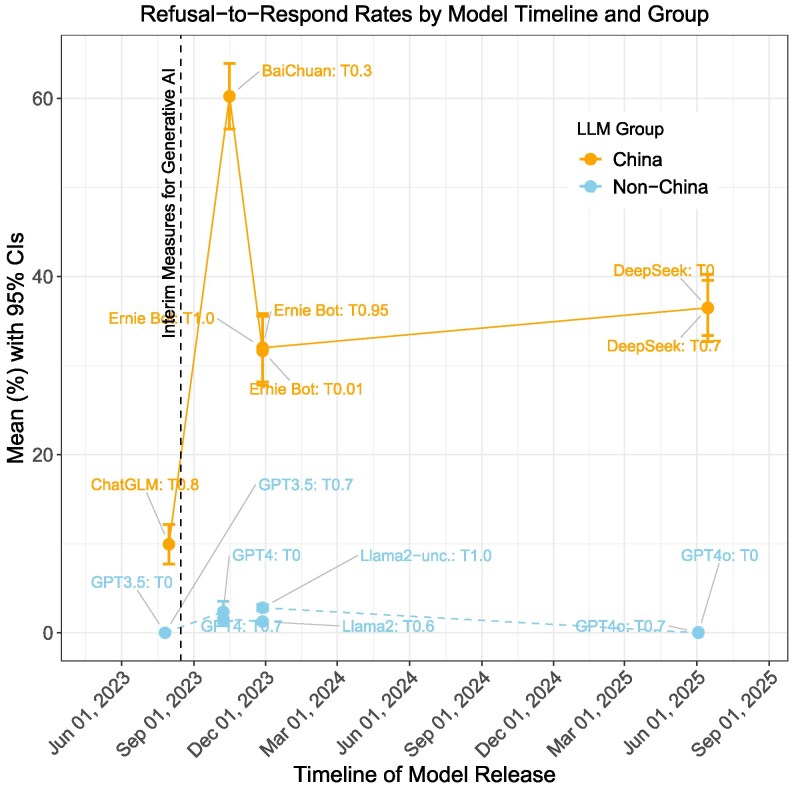
Refusal to respond rate over time for China and non-China models.

One of the China models we examined, ChatGLM, is based on a version released before China’s Measures took effect on 2023 August 15. Figure 6 plots the refusal-to-respond rate by model release timeline and group. As shown in the figure, ChatGLM exhibits a much lower refusal-to-respond rate compared to China-based models released after the Measures, while non-China models show little difference before and after this regulatory change. Notably, ChatGLM also exhibits the longest average response length (Fig. 2) and next to lowest rates of complete inaccuracy (Fig. 3). While this pattern is suggestive of regulatory influence, it does not constitute an identification strategy and other factors, such as the academic origins and orientation of ChatGLM, may also explain its lower level of censorship.

## Discussion

Our results reveal important differences between China-originating models and non-China-originating models, suggesting that state intervention may play a role in shaping political biases in China LLMs. In particular, we find that these differences cannot be fully explained by contextual factors affecting model development, market conditions, or technical constraints.

However, several limitations should be kept in mind when interpreting these findings. First, while we show that alternative explanations cannot alone account for the patterns we observe, we cannot rule out their partial contributions. General model tendencies, characteristics of the training data, and engineering choices still influence outputs. Second, our study is observational and cross-sectional; it does not establish a causal linkage between regulatory pressures and censorship behaviors of China-based LLMs. Third, this study is not designed to examine how linguistic framing might influence model responses. Prior research finds that LLMs are sensitive to prompt wording (39). For example, describing Ai Weiwei as a “pro-democracy activist” vs. an “artist” may affect observed censorship patterns. In this study, prompts are held constant across models. Fourth, our analysis relies on API-level prompting, which may not fully capture how end users experience these systems through consumer-facing interfaces. In manual tests, we identified censorship at three levels: response, conversation, and account. While the current study focuses on the response level, it does not address cases where entire conversation threads are shut down and deleted,^w^ nor does it consider potential account suspensions after repeated blocked queries.

As the number of users relying on LLMs rapidly grows, and as applications built on these models grow in influence, our findings have implications for how censorship by China-based LLMs may shape users’ access to information and their very awareness of being censored. While many of the prompts we analyze are politically sensitive, not all are, highlighting how censorship-induced biases can restrict even general political knowledge that emerges from routine or curiosity-driven inquiries. The consequences depend on who is asking. Users who are already politically informed may pose sensitive questions largely to test the system, learning little that is new. By contrast, less aware users often turn to LLMs seeking factual background or explanations. For these individuals, refusals or inaccurate outputs directly impede knowledge acquisition. Moreover, as LLMs increasingly underpin commercial applications such as search, virtual assistants, and content creation, users may unwittingly encounter censorship. For example, when we asked DeepSeek for travel advice about the Mutianyu section of the Great Wall, the model refused to respond, possibly due to nearby rock inscriptions praising Mao Zedong, thereby filtering a seemingly apolitical tourism query. Unlike traditional forms of censorship that involve outright content removal or blocking access, LLM-based censorship typically involve some kind of reply—such as an apology or justification for not answering or even inaccurate information—making the suppression of information less obvious. This subtlety could make it more difficult for people to recognize when censorship is occurring, quietly shaping perceptions, decision-making, and behaviors.

These findings also highlight the potential for the Chinese government to extend its information control efforts through LLMs, shaping what information is accessible to the public in China and further consolidating its control over information flows. This influence may extend beyond China’s borders. Companies outside of China building applications on Chinese-developed foundation models could inadvertently propagate censorship.^x^ In addition, individuals outside of China who interact with models using Chinese may also encounter biased outputs.

Our curated set of 145 prompts can serve as a benchmark for future research on LLM censorship in China, though scholars should update and expand these questions to capture evolving contexts (all prompts are detailed in the SI S1). Altogether, these results underscore the importance of understanding the influence of state and government regulations on foundation models, and the implications for knowledge, discourse, and access to information amid the expanding role of AI in shaping human and societal interactions.

## Supplementary Material

pgag013_Supplementary_Data

## Data Availability

The data underlying this article are available at the Harvard Dataverse at https://dataverse.harvard.edu/dataset.xhtml?persistentId=doi:10.7910/DVN/VQMOJU.

## Notes

a In this article, we focus on LLMs that are foundation models, AI systems trained on massive amounts of internet data adaptable across a wide variety of tasks.

b Examples include UK’s Data Protection and Digital Information Bill, Brazil’s Bill No. 2338/2023, and India’s Digital Personal Data Protection Bill (2023).

c See https://www.cac.gov.cn/2023-07/13/c_1690898327029107.htm for original text, https://www.chinalawtranslate.com/en/generative-ai-interim/ for translation.

d See https://www.cac.gov.cn/2025-01/08/c_1738034725920930.htm.

e See https://carnegieendowment.org/research/2025/07/chinas-ai-policy-in-the-deepseek-era.

f See https://www.cac.gov.cn/2025-04/30/c_1747719097461951.htm

g See “1. Beijing’s ‘Clean Up the Internet and Rectify the Abuse of AI Technology’ campaign has achieved initial results” (1.北京市“清朗·整治AI技术滥用”专项行动取得初步成效) tab of https://www.cac.gov.cn/2025-06/20/c_1752129980667315.htm.

h Use based on consumer adoption as well as commercial applications.

i “Less-sensitive topics” exclude topics related to dissidents and repressive events.

j Among the 145 questions, 30 are what we consider less-sensitive topics.

k For Ernie Bot, we were not able to obtain 100 conversations at each temperature.

l BaiChuan at 0.3, ChatGLM at 0.8, Llama2 at 0.6, Llama2-uncensored at 1.0.

m Ernie Bot at 0.01, 0.95, and 1.0; GPT3.5 at 0 and 0.7; GPT4 at 0 and 0.7.; GPT4o at 0 and 0.7; Deepseek at 0 and 0.7.

n Refusal keywords include “抱歉” (sorry), “我无法” (I am unable), “我不能” (I cannot), “我没有” (I do not have), “我不知道” (I do not know), and “对不起” (sorry). This approach achieves over 90% agreement with human and LLM evaluations of nonresponse. We chose this method rather than machine labeling of nonresponse for simplicity and transparency.

o Some models generate internal reasoning when prompted via API. We exclude any reasoning-phase text from the length calculation so that what users actually see is counted.

p Completely inaccurate responses can stem from content-filtering and blocking as well as from hallucinations when the model has limited training data and sparse knowledge.

q Analysis is based on Chinese-language benchmark responses and attains 83% agreement with human accuracy judgments. We exclude Llama from this analysis because some of its Chinese-prompt outputs appear in English, and back-translation can introduce errors.

r For refusal to respond for different topics, see SI S2.4.

s We do not believe these differences are due to training data cut-off dates, since newer China models still show clear divergence from non-China models.

t In addition to affecting training data, these factors can also affect RLHF.

u To facilitate comparisons between English and Chinese, we translate English responses to Chinese with GPT-4o. Human review of a 5% sample, stratified by model, found all translations to be accurate.

v We also find similar response lengths between China and non-China models for the 30 less-sensitive topics (see SI S2.2), which contrasts with the pattern observed across all topics. This further suggests that the observed censorship patterns are unlikely to be driven solely by contextual factors, company objectives, or technical capabilities.

w For example, on Ernie Bot, certain prompts trigger the message: “Why don’t you change the topic and restart; starting new conversation” (换个话题重新开始吧, 新建对话), after which the previous conversation is erased from the chat history.

x For example, concerns about GeoGPT, built on Alibaba’s Qwen, stem from this issue, see https://www.theguardian.com/technology/article/2024/jun/24/geologists-censorship-bias-chinese-chatbot-geogpt.
